# Supplementary material for: Hyperphosphatemia Contributes to Skeletal Muscle Atrophy in Mice
Source: Int J Mol Sci. 2024 Aug 28;25(17):9308. doi: 10.3390/ijms25179308 (PMC11395169; doi:10.3390/ijms25179308)
Supplement: Supplementary file 1 [file ijms-25-09308-s001.zip › ijms-3141664-supplementary.pdf]

Supplementary Figures:

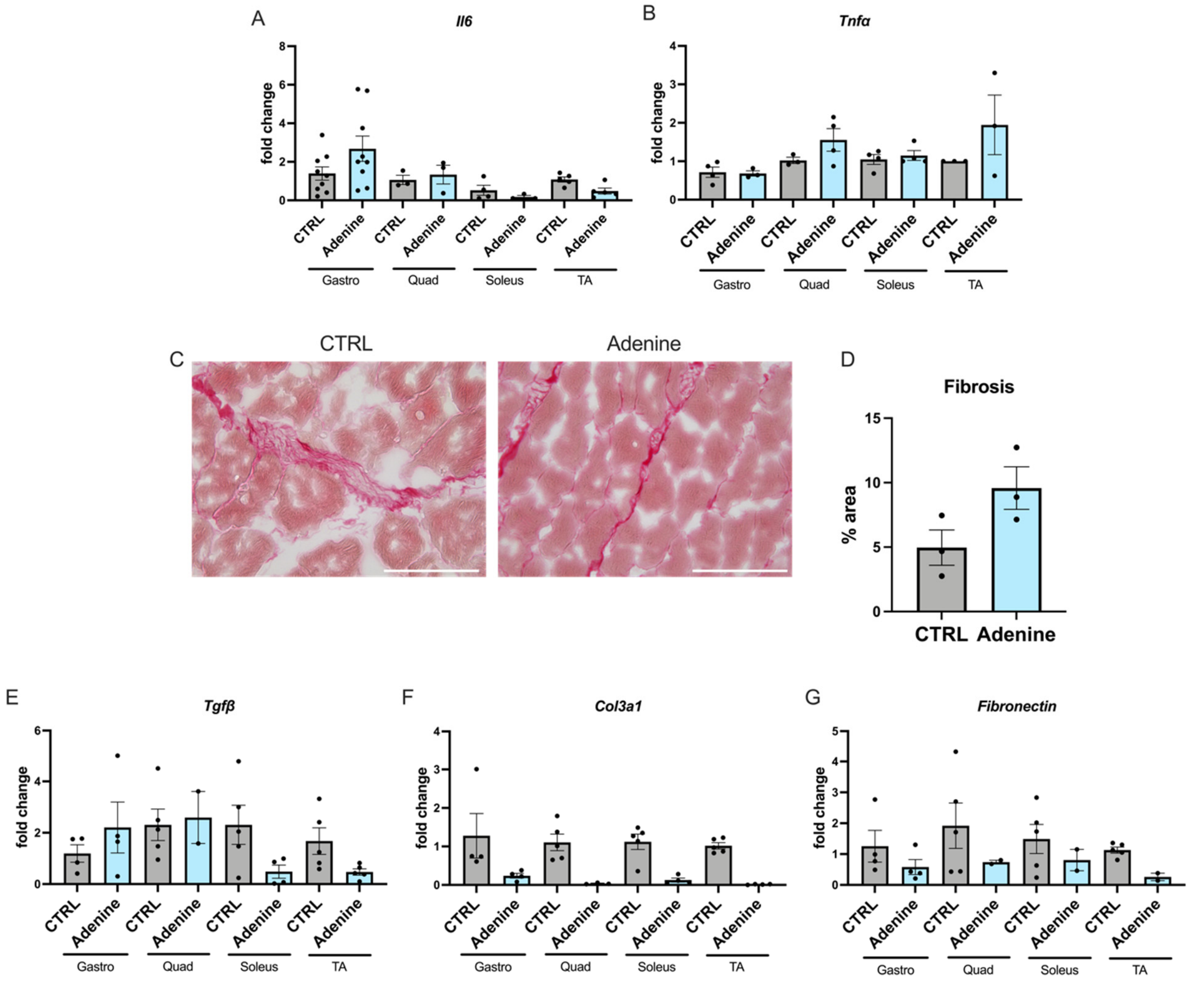

Figure S1: Mice receiving an adenine-rich diet do not develop skeletal muscle inflammation or fibrosis. We analyzed C57BL/6J mice that received an adenine-rich or control (CTRL) diet for 14 weeks. (**A, B**) qRT-PCR expression analysis of gastrocnemius (gastro), quadricep (quad), soleus, and tibialis anterior (TA) muscles for *Interleukin 6* (*Il6*) or *Tumor necrosis factor alpha* (*TNFα*) (*n*=3-9). (**C**) Representative images of gastrocnemius muscle sections stained with Picrosirius red to visualize collagen fibers (scale bar = 100 µm). (**D**) Quantification of the fibrotic area based on Picrosirius red staining of gastrocnemius sections (*n*=3). (**E-G**) Expression analysis of gastro, quad, soleus, and TA by qRT-PCR for *Transforming growth factor beta* (*Tgfβ*), *Collagen type III alpha 1 chain* (*Col3a1*) and *Fibronectin* (*n*=2-5). Comparison between CTRL versus adenine mice was performed in an unpaired two-tailed t-test, or a one-way ANOVA followed by a post-hoc Tukey's test. All values are mean ± standard error of the mean (SEM).

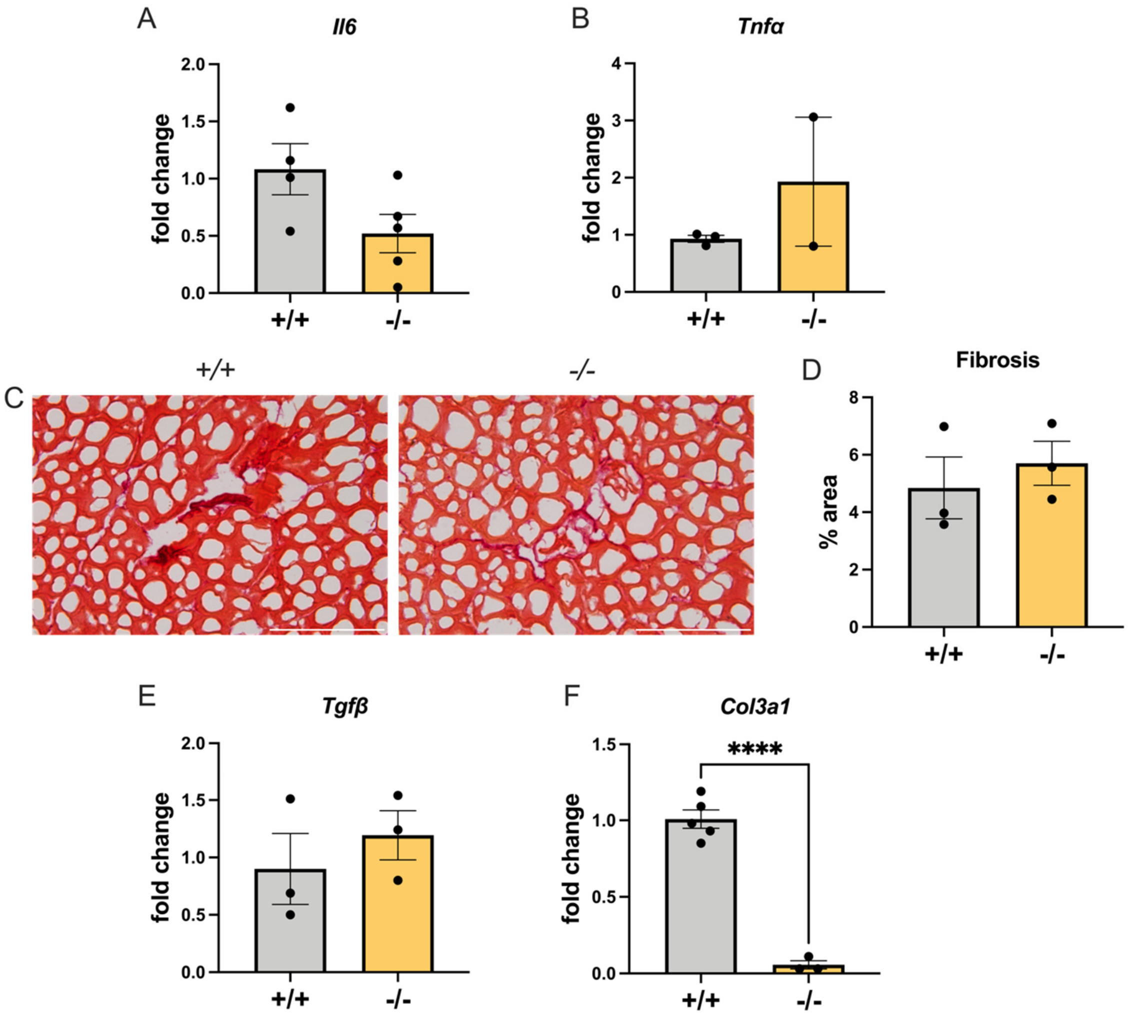

Figure S2: *Col4α3*$^{-/-}$ mice do not develop skeletal muscle inflammation or fibrosis. We analyzed mice with global deletion of *Collagen type IV alpha 3 chain* (-/-) and wildtype littermates (+/+) at 10 weeks of age. (**A, B**) qRT-PCR expression analysis of gastrocnemius muscle for *Interleukin 6* (*Il6*) and *Tumor necrosis factor alpha* (*TNFα*) (*n*=2-5). (**C**) Representative images of gastrocnemius muscle sections stained with Picrosirius red to visualize collagen fibers (scale bar = 100 µm). (**D**) Quantification of the fibrotic area based on Picrosirius red staining of gastrocnemius sections (*n*=3). (**E, F**) Expression analysis of gastrocnemius by qRT-PCR for *Transforming growth factor beta* (*TGFβ*) and *Collagen type III alpha 1 chain* (*Col3a1*) (*n*=3-5; ****p<0.0001). Comparison between *Col4a3*$^{+/+}$ versus *Col4a3*$^{-/-}$ mice was performed in an unpaired two-tailed t-test. All values are mean ± standard error of the mean (SEM).

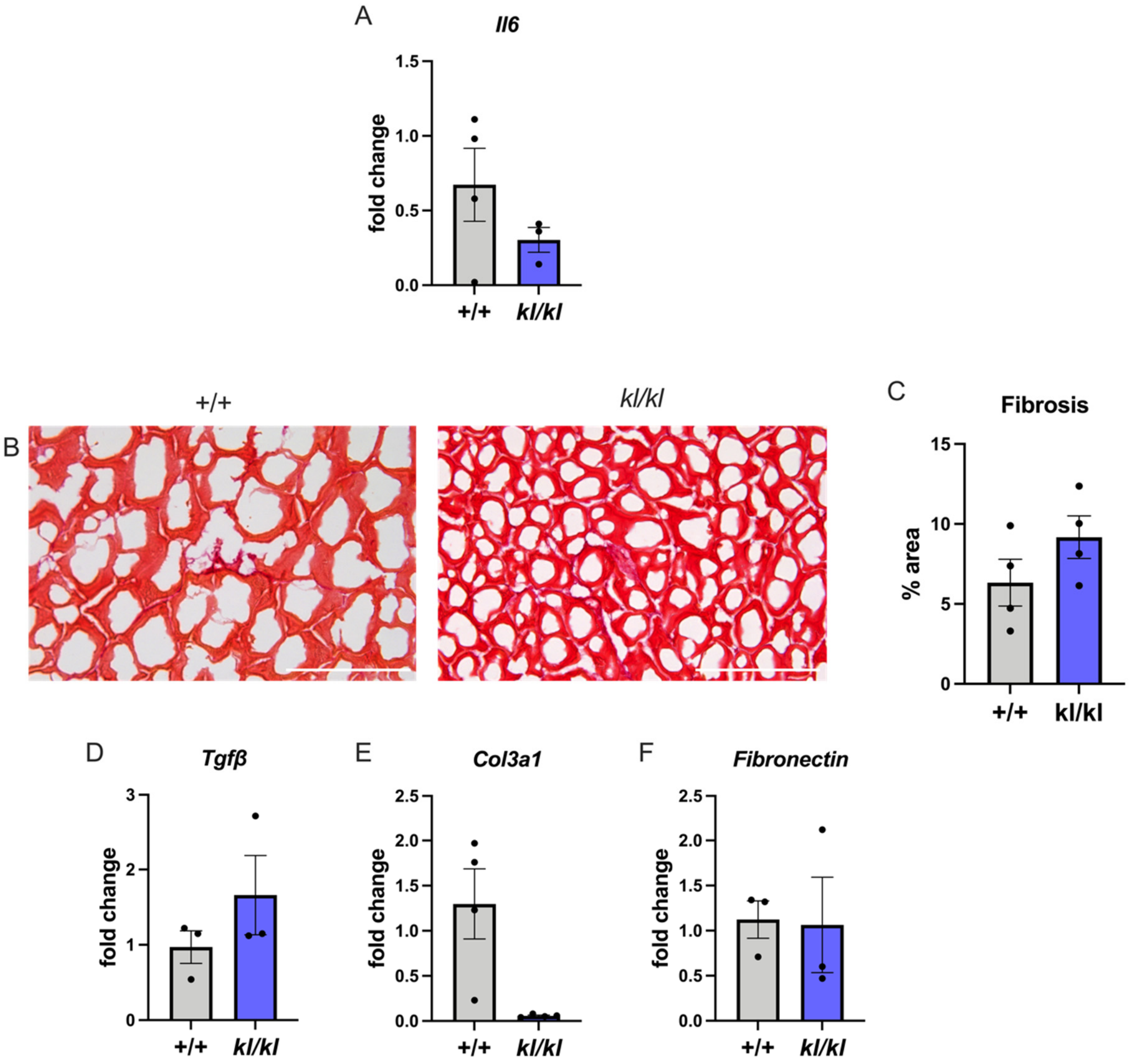

Figure S3: *Klotho*-deficient mice do not develop skeletal muscle inflammation or fibrosis. Homozygous *klotho*-deficient mice (*kl/kl*) and wildtype littermates (+/+) were analyzed at 8 weeks of age. (**A**) qRT-PCR expression analysis of gastrocnemius muscle for *Interleukin 6* (*Il6*) (*n*=3-4). (**B**) Representative images of gastrocnemius muscle sections stained with Picrosirius red to visualize collagen fibers (scale bar = 100 µm). (**C**) Quantification of the fibrotic area based on Picrosirius red staining of gastrocnemius sections (*n*=4). (**D-F**) Expression analysis of gastrocnemius by qRT-PCR for *Transforming growth factor beta* (*Tgfβ*), *Collagen type III alpha 1 chain* (*Col3a1*) and *Fibronectin* (*n*=3-4). Comparison between +/+ versus *kl/kl* mice was performed using an unpaired two-tailed t-test. All values are mean ± standard error of the mean (SEM).

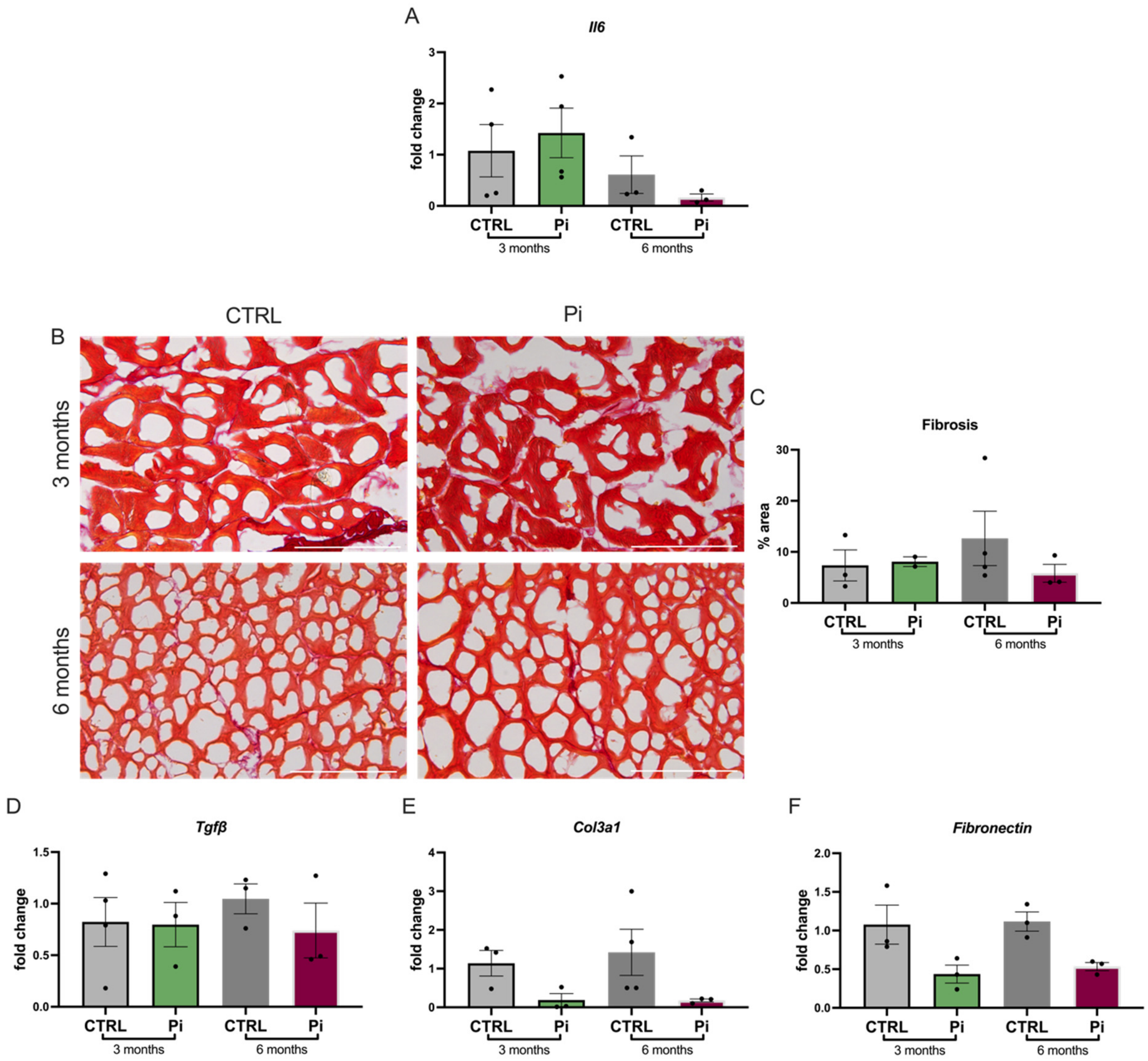

Figure S4: Mice receiving a high-phosphate diet do not develop muscle inflammation or fibrosis. We analyzed C57BL/6J mice that received a 3% phosphate (Pi) or control (CTRL) diet for 3 or 6 months. **(A)** qRT-PCR expression analysis of gastrocnemius muscle for *Interleukin 6* (*Il6*) (*n*=3-4). (**B**) Representative images of gastrocnemius muscle sections stained with Picrosirius red to visualize collagen fibers (scale bar = 100 μm). (**C**) Quantification of the fibrotic area based on Picrosirius red staining of gastrocnemius sections (*n*=2-4). (**D-F**) Expression analysis of gastrocnemius by qRT-PCR for *Transforming growth factor beta* (*TGFβ*), *Collagen type III alpha 1 chain* (*Col3a1*) and *Fibronectin* (*n*=3-4). Comparison between 3-month of CTRL and Pi or between 6-month of CTRL and Pi was performed using an unpaired two-tailed t-test, or a one-way ANOVA followed by a post-hoc Tukey's test. All values are mean ± standard error of the mean (SEM).

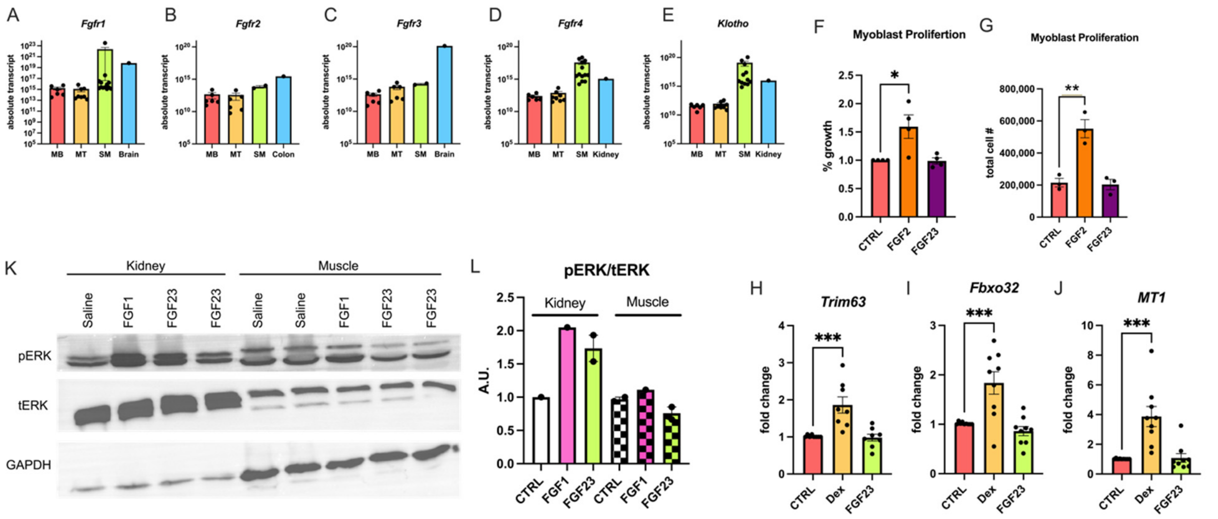

Figure S5: FGF23 elevations do not affect C2C12 myoblasts or myotubes or mouse skeletal muscle tissue. (**A-E**) Total RNA was isolated from cultured C2C12 myoblasts (MB), cultured C2C12 myotubes (MT), and gastrocnemius muscle isolated from C57BL/6J mice (SM). Mouse kidney, brain and colon were used as positive controls. Absolute transcript numbers were determined by qRT-PCR for *Fibroblast growth factor receptors* (*FGFR*) *1-4* and *Klotho* (*n*=2-13). (**F**) Alamar blue dye was added to proliferating C2C12 myoblasts that were cultured in normal medium (CTRL) or in the presence of 25 ng/mL of FGF2 or FGF23. After 24 hours, absorbance was determined using a plate reader (*n*=4; *p<0.05, **p<0.001. (**G**) C2C12 myoblasts were cultured in normal medium (CTRL) or in the presence of 25 ng/mL of FGF2 or FGF23 for 24 hours, stained with trypan blue and counted (*n*=3; ***p<0.001). (**H-J**) C2C12 myotubes were cultured in the presence of normal medium (CTRL), 100 µM dexamethasone (Dex), or 25 ng/mL FGF23 for 24 hours. qRT-PCR expression analysis for *Tripartite motif containing 63* (*Trim63*), *F-box protein 32* (*Fbxo32*), and *Metallothionein 1* (*MT1*) (*n*=8-9; ***p<0.001). (**K**) C57BL/6J mice were injected with 200 µL of saline, 5 µg of FGF1 solved in 200 µL of saline, or 5 µg of FGF23 solved in 200 µL of saline. After 20 minutes, kidney and skeletal muscle tissue were isolated for protein extraction and Western blot analysis. Images of representative Western blots for phosphorylated extracellular signal-regulated kinase (pERK) and total extracellular signal-regulated kinase (tERK), as well as GAPDH used as a loading control. (**L**) Quantification of Western blot signals of pERK in relation to tERK by densitometry (*n*=1-2). Comparison between cell treatment groups was performed using a one-way ANOVA followed by a post-hoc Tukey's test. All values are mean ± standard error of the mean (SEM).

Supplementary Tables:

Table S1: Mouse primer sequences

Primer Name	Forward Sequence	Reverse Sequence
Gapdh	CCAATGTGTCCGTCGTGGATCT	GTTGAAGTCGCAGGAGACAACC
Fbxo32	TGAGCGACCTCAGCAGTTAC	GCGCTCCTTCGTACTTCCTT
Trim63	GAGGGCCATTGCTTTGGGA	TGGTGTTTCTTCTTTACCCTCTGT
MT1	CGACTTCAACGTCCTGAGTAC	AGGAGCTGGTGCAAGTG
Mstn	CTCCAGAATAGAAGCCATA	GCAGAAGTTGTCTTATGC
Il6	CTCTGGGAAATCGTGGAAAT	CCAGTTTGGTAGCATCCATC
Tgfβ	CTGCTGACCCCCACTGATAC	AGCCCTGTATTCCGTCTCCT
Tnfα	ATGGCCTCCCTCTCATCAGT	TGGTTTGCTACGACGTGGG
Fibronectin	CAGACAATGCCGTGGTCCTA	GTTGGGGAATCGAGACCTGT
Collagen type 3a1	TGACTGTCCCACGTAAGCAC	GAGGGCCATAGCTGAACTGA
Klotho	TGTATGTGACAGCCAATGGAATCG	GAATACGCAAAGTAGCCACAAAGG
Fgfr1	GCTTGACGTCGTGGAACGAT	AGCCACTGAATGTGAGGCTG
Fgfr2	ATCCCCCTGCGGAGACA	GAGGACAGACGCGTTGTTATCC
Ffgr3	GTGTGCGTGTAACAGATGCTC	CGGGCGAGTCCAATAAGGAG
Fgfr4	TGAAGAGTACCTTGACCTCCG	TCATGTCGTCTGCGAGTCAG
